# Mom, dad, put down your phone and talk to me: how parental phubbing influences problematic internet use among adolescents

**DOI:** 10.1186/s40359-024-01620-0

**Published:** 2024-03-05

**Authors:** Saifang Liu, Peiqian Wu, Xiaoxi Han, Mengyun Wang, Yuecui Kan, Kuiyuan Qin, Jijun Lan

**Affiliations:** 1 School of Psychology, Shaanxi Normal University, 199 South Chang’an Road, 710061 Xi’an, China; 2 Shaanxi Provincial Key Laboratory of Behavior and Cognitive Neuroscience, 710061 Xi’an, China; 3 School of Educational Science, Anhui Normal University, 241000 Wuhu, China; 4 Department of Medical Psychology, Psychological Science and Health Management Center, Harbin Medical University, Harbin, China

**Keywords:** Parental phubbing, Problematic internet use, Parent–child relationship, Basic psychological needs satisfaction, Adolescents

## Abstract

**Background:**

The positive association of parental phubbing with internalising and externalising problems among adolescents has gained academic traction. To date, limited research has investigated the association of parental phubbing and adolescents’ Problematic Internet Use (PIU). Furthermore, the mechanism underlying this association is largely unknown. These gaps limit our understanding of family-related issues affecting PIU among adolescents. The present study explores whether there is a relation between parental phubbing and PIU and investigates the mechanisms underlying this relation among adolescents.

**Methods:**

The participants were 495 junior high schoolers aged 11–15 years. Participants completed questionnaires on their experiences with PIU, parental phubbing, parent–child relationships, and basic psychological needs satisfaction.

**Results:**

The results showed a direct and indirect positive association between parental phubbing and PIU. Furthermore, parental phubbing indirectly influenced PIU and was mediated by the parent–child relationship and basic psychological needs satisfaction, respectively. Moreover, the parent–child relationship and basic psychological needs satisfaction were sequentially mediated.

**Conclusions:**

Our study highlights the crucial role of parents in the development of adolescent PIU and provides theoretical and practical guidelines for PIU prevention and intervention.

**Supplementary Information:**

The online version contains supplementary material available at 10.1186/s40359-024-01620-0.

## Background

Problematic Internet Use (PIU) refers to an individual’s loss of control over their Internet use [1]. With the increasing popularity of Internet use, PIU among adolescents has become a global social problem. Adolescence refers to the period in which individuals transition from childhood to adulthood. Adolescents gradually shed their parents’ supervision and begin making decisions independently. However, owing to their weak self-control, they are at risk of developing problematic behaviours, such as PIU [2]. For instance, previous researchers’ work reported that there was a prevalence of 33% for PIU among Spanish adolescents [3]. A survey in Egypt has found that 35.1% of adolescents suffer from moderate Internet addiction, and 3.6% suffer from severe addiction [4]. Several studies have suggested there are harmful effects of PIU on adolescents’ physiological, physical, psychological, and behavioural development, such as gray matter abnormalities, vision loss, depression, and sleep disturbances [5–8]. Given PIU’s severity and negative consequences, policymakers, researchers, and school administrators have increased focus on PIU among adolescents.

A central issue in the academic research on PIU is determining influences in the development of PIU in adolescents. Previous research has identified some predictors of PIU in adolescents, such as personality traits [9, 10], interpersonal relationship quality [11], and mental symptoms [12]. Based on Bronfenbrenner’s socioecological model, adolescents’ behaviours are impacted by various levels of ecological systems, of which family is the closest environment [13]. Thus, the family environment likely has the most impact on adolescents’ behaviour. Consistent with this view, previous studies have shown that family-related factors play an important role in the onset of adolescent PIU [14, 15].

In the current information age, the mobile phone provides convenience to users. However, this trend has brought attention to parental phubbing, a negative element in the family environment. Phubbing is a type of social exclusion and interpersonal neglect [16]. Parental phubbing is a phenomenon in which parents ignore their children while paying attention to smartphones [17]. Limited research has investigated the association of parental phubbing and adolescents’ PIU [18, 19]. Furthermore, the mechanism underlying this association is largely unknown. These gaps limit our understanding of the impact of family-related issues on adolescents’ PIU. From an evolutionary perspective, the parent–child relationship is negatively influenced by parental exclusion and neglect [20] and is a predictor of PIU [21]. Additionally, basic psychological needs are important psychological nutrients in an individual’s development [22], and the obstruction of needs fulfilment can lead to problematic behaviours, such as PIU [23]. Studies have shown that family factors, such as parenting style and the parent–child relationship, play an important role in satisfying adolescents’ basic psychological needs [22]. Hence, in this study, we investigate whether there is a relation between parental phubbing and PIU and how the parent–child relationship and basic psychological needs satisfaction (BPNS) account for that relation. These insights may help researchers and educators gain a deeper understanding of how parental phubbing affects PIU and enable family therapists to effectively help adolescents who experience parental phubbing overcome PIU.

### The relationship between parental phubbing and PIU

Parental phubbing may induce behavioural problems in adolescents, such as interpersonal aggression [24] and prosocial behaviour [25]. Similarly, some researchers have suggested that parental phubbing may lead to problematic smartphone use among adolescents [26–30]. According to social learning theory, individuals’ behaviours can be learned and strengthened by observing the behaviours of others, such as family members [31]. Therefore, parents’ habit of frequently focusing on their smartphones may be imitated by their children. As adolescents with problematic smartphone use tend to be attracted to the network functions of mobile phones (e.g., online gaming, short-form videos, and social media), we speculate that parental phubbing may be positively correlated with adolescents’ PIU.

Additionally, previous literature has suggested that parental phubbing may have negative outcomes, such as poor parent–child relationships and the hindrance of basic psychological needs [32, 33]. It has been suggested that individuals with negative experiences are more likely to develop PIU [34, 35]. Therefore, the influence of parental phubbing on PIU may be both direct and indirect.

## The parent–child relationship as a mediator

The parent–child relationship is defined as the quality of the connections between parents and their children, which plays a crucial role in an adolescent’s development [36]. Generally, a negative parent–child relationship may increase the risk of developmental maladjustment in children and adolescents, leading to emotional and behavioural problems [11, 37, 38]. Additionally, parents serving as the foundation of children’s upbringing are important factors influencing parent–child relationships. Therefore, the parent–child relationship has been examined as a mediator linking parent-related factors (e.g., socioeconomic status, active parental mediation, and parents’ response to children’s performance) to adolescent outcomes [39, 40].

Following a review of the previous literature, we hypothesised that the parent–child relationship acts as a mediator linking parental phubbing to adolescent PIU. The quality of parent–child relationships may be weakened in the context of parental phubbing. From an evolutionary perspective, providing feedback to close figures (i.e., being responsive) is important for establishing and maintaining close relationships [20]; yet, phubbing in front of family members and friends implies neglect [41]. Thus, interpersonal relationships can be negatively affected by phubbing. Consistent with this view, empirical studies have found that phubbing is associated with low-quality social interactions between communicators and low relationship satisfaction between partners or between employers and employees [16, 42]. Similarly, recent studies have demonstrated the negative link between parental phubbing and the parent–child relationship [30, 43, 44]. Moreover, a dysfunctional parent–child relationship plays a significant role in the development and severity of PIU in adolescents [21]. Scholars have reported that the parent–child relationship is negatively associated with PIU [37, 45]. For instance, a study indicates that that when adolescents feel that their emotional connection with their parents is weak, they are more likely to engage in deviant behaviours, such as PIU [45]. Based on this, we propose that parental phubbing may affect PIU through the parent–child relationship.

## Satisfaction of basic psychological needs as a mediator

Self-determination theory (SDT) proposes that the motivation of all individuals comprises three types of innate basic psychological needs: competence, autonomy, and relatedness [22]. The BPNS is characterized as the extent to which an individual’s external situation satisfies their basic psychological needs. A frustrating or rejecting environment can hinder BPNS, which is more likely to result in an individual facing difficulty adapting to their circumstances. This can lead to a strong urge to gain or maintain the desired level of need satisfaction in other areas, such as the Internet [46–48].

In the present study, we speculate that BPNS, as an important intrinsic motivation, may be an important mediator of the association between parental phubbing and PIU among adolescents. Parental phubbing may interfere with BPNS. Previous research has suggested that parental phubbing is a rejecting family environment [18, 24, 49]. According to SDT, an unsupportive or rejecting environment can hinder BPNS [22]. Directly, several studies have suggested that parental phubbing is a negative predictor of BPNS [49, 50]. For example, researchers have used a longitudinal design to demonstrate that parental phubbing hinders the satisfaction of relatedness needs among adolescents [50]. Additionally, existing empirical evidence suggests that the characteristics of the Internet can satisfy adolescents’ needs for autonomy, competence, and relatedness [51–53]. For example, researchers have conducted four studies to confirm that the use of Facebook can help individuals meet their relatedness needs [52]. However, compared to adults, adolescents have weaker self-control, which may make them more susceptible to PIU [54]. Based on the existing literature, it is likely that the BPNS mediates the relationship between parental phubbing and PIU.

## Roles of the parent–child relationship and BPNS in linking parental phubbing to PIU

To date, no study has examined the cooperative relationship between the parent–child relationship and BPNS in linking parental phubbing to PIU. We aim to fill this gap with the current study. According to SDT, a supportive environment can facilitate individuals’ natural growth processes by meeting their basic psychological needs [46]. Previous literature suggests that adolescents with a positive parent–child relationship perceive emotional warmth and social support, and adolescents are able to recognise a lack of social support in poor-quality parent–child relationships [55]. Thus, a positive parent–child relationship may be a protective factor in BPNS. The empirical literature has shown that the parent–child relationship is positively related to BPNS [56–58]. For example, a study on left-behind children has found that parent–child cohesion is significantly positively related to BPNS [56]. Understanding that parental phubbing may be an antecedent of the parent–child relationship and that BPNS may be an antecedent of PIU in adolescents, the current study assumes that the parent–child relationship and BPNS sequentially mediate the relationship between parental phubbing and PIU.

## Methods

### Participants

The required sample sizes for the multiple mediator model applied in this study were calculated prior to data collection using the tool provided by Schoemann et al. [59]. Based on previous related studies in this area, correlations of *r* = 0.20 (*SD* = 0.10) were assumed between the independent variable, the two serial mediators, and the dependent variable. To reach a power of 0.80 for the indirect effects of the two serial mediators, 485 participants were needed. Therefore, this study recruited participants from three Chinese secondary schools in Shanxi Province, China, and conducted the study in classroom settings. A total of 510 adolescents volunteered to participate in this study during school hours. It was emphasised that the students’ participation was voluntary and confidential. The researcher distributed a total of 510 self-report paper-and-pencil questionnaires, of which 495 were validated (97.1%). The participants were 11–15 years old (mean age = 13.39 ± 0.77 years; 56.2% girls). Informed consent was obtained from participants, their parents, and their teachers before data collection, and approval was obtained from the Ethics Review Committee of the first author’s university. When the participants finished responding to the survey, we gave 2 yuan to each participant as a reward.

### Measures

#### Internet Addiction Diagnostic Questionnaire

The Chinese version of the Internet Addiction Diagnostic Questionnaire (Cronbach’s alpha = .84 for this study) was used to measure PIU in the participants [35, 60]. The version used contains 10 items, with each item scored on a Likert scale ranging from 1 (‘not at all true’) to 6 (‘always true’). Confirmatory factor analysis (CFA) showed the data fit well: χ2/df = 2.37, CFI = 0.95, TLI = 0.94, SRMR = 0.04. The average score for all items was calculated, with higher scores indicating higher levels of PIU.

#### Basic Psychological Needs Scales

The BPNS was measured by the Basic Psychological Needs Scale [46], which consists of 21 items divided into three dimensions based on needs: competence, autonomy, and relatedness (Cronbach’s alpha = .84 for this study). Each item was scored on a scale from 1 (‘not at all true’) to 7 (‘always true’). The fit indices of CFA for the Basic Psychological Needs Scale were acceptable: χ2/df = 2.26, CFI = 0.87, TLI = 0.86, SRMR = 0.05. The average score for all items was calculated as an estimate of the overall BPNS.

#### Parental Cohesion Scale

The parent–child relationship was measured using the Chinese version of the 20-item Parental Cohesion Scale (Cronbach’s alpha = 0.85 for this study), which comprises two dimensions: father–child cohesion and mother–child cohesion [61]. Each item is scored on a Likert scale ranging from 1 (‘nearly never’) to 5 (‘almost always’). The fit indices of CFA for the Parental Cohesion Scale were acceptable: χ2/df = 3.85, CFI = 0.91, TLI = 0.89, SRMR = 0.06. The average score of the two dimensions represents the overall parent–child relationship score. Higher average scores indicate a higher-quality parent–child relationship.

#### Parental Phubbing Scale

The present study adopted the Chinese version of the Parental Phubbing Scale (Cronbach’s alpha = .74 for this study) to measure parental phubbing [62]. The respective assessment included five items, with each item rated on a five-point Likert scale ranging from 1 (‘never’) to 5 (‘always’). The CFA index suggested a good fit: χ2/df = 3.07, CFI = 0.93, TLI = 0.90, SRMR = 0.04. The average score indicated the severity of parental phubbing.

### Analyses

All analyses were performed using SPSS 22.0. First, common method bias (CMV) was assessed. Then, bivariate correlations among all variables were calculated, given that CMV was not revealed in the present data. Thereafter, multiple mediation analyses were performed to test the indirect effects using SPSS macro-PROCESS. The mediation effects of the parent–child relationship and BPNS were considered significant if the 95% CI for the index of multiple mediations did not include zero [63].

## Results

### Testing for common method bias

To improve the precision of our results, three methods were used to control for CMV: changing the name of the questionnaire to reduce predictability, scoring some of the items in reverse, and balancing the order of different dimensions. Additionally, CMV was tested using Harman’s one-factor test. Based on the results of exploratory factor analysis, 16 factors with eigenvalues above one were extracted. The first factor explained only 14.50% of the variance of all variables, which is less than the suggested trade-off of 40% [64]. Thus, there is no serious CMV in this study.

### Preliminary analyses

Table 1 shows the descriptive statistics and correlations for all variables. The results suggested positive associations between parental phubbing and PIU as well as between the parent–child relationship and BPNS. Parental phubbing was negatively associated with the parent–child relationship and BPNS. Furthermore, the parent–child relationship and BPNS were found to have a negative impact on PIU.

**Table 1:** Means, standard deviations, and bivariate correlations of the main variables

Variables	*M*	*SD*	1	2	3	4
1. Parental phubbing	2.68	0.62	-			
2. Parent–child relationship	3.55	0.54	− 0.26***	-		
3. BPNS	4.51	0.90	− 0.23***	0.30***	-	
4. PIU	2.51	0.99	0.27***	− 0.23***	− 0.27***	-

Note ***p <.001; PIU: Problematic Internet Use; BPNS: Basic Psychological Needs Satisfaction; M: Mean; SD: Standard Deviation

### Testing the multiple mediation model

**Fig. 1:**
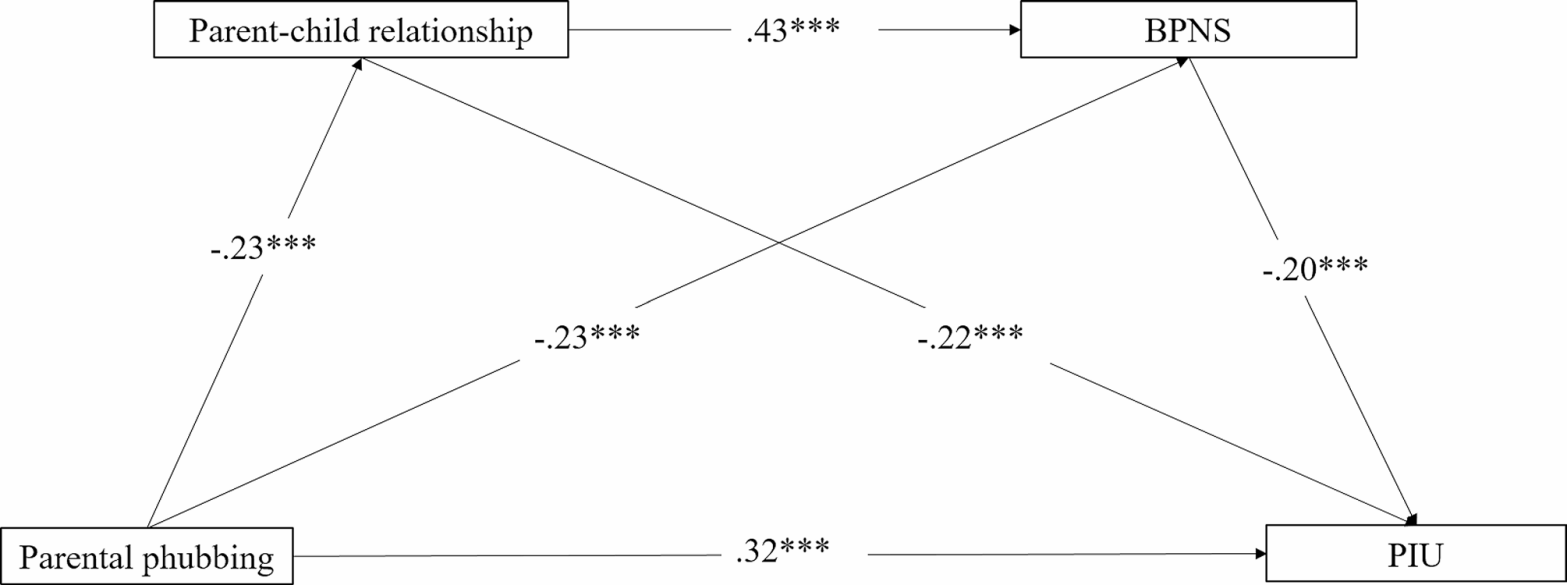
The multiple mediation model. Path values show path coefficients. ***p <.001; **p <.01; PIU: Problematic Internet Use; BPNS: basic psychological needs satisfaction

First, regression analysis revealed that parental phubbing was positively associated with PIU (*B* = 0.32; *p* <.001). Furthermore, when both the parent–child relationship and BPNS were included in the regression equation, all direct effects among the main variables of interest were found to be statistically significant (see Table 2; Fig. 1). Specifically, parental phubbing was found to be positively associated with PIU (*B* = 0.32, *p* <.001), whereas it was negatively associated with the parent–child relationship (*B* = − 0.23, *p* <.001) and BPNS (*B* = − 0.23, *p* <.001). Moreover, the parent–child relationship was positively associated with BPNS (*B* = 0.43, *p* <.001), whereas it was negatively associated with PIU (*B* = − 0.22, *p* <.001). BPNS was found to be negatively associated with PIU (*B* = − 0.20, *p* <.001).

**Table 2:** Testing the pathways of the multiple mediation model

Path	Point estimate	95% CI	
		Lower	Upper
**a. Total effects model**			
Parental phubbing → PIU	0.43***	0.30	0.57
**b. Multiple mediation model**			
**Direct effects**			
Parental phubbing → PIU	0.32***	0.18	0.46
Parental phubbing → Parent–child relationship	− 0.23***	− 0.30	− 0.15
Parent–child relationship → PIU	− 0.22***	− 0.38	− 0.06
Parental phubbing → BPNS	− 0.23***	− 0.36	− 0.11
BPNS → PIU	− 0.20***	− 0.30	− 0.11
Parent–child relationship → BPNS	0.43***	− 0.29	− 0.58
**Indirect effects**			
Parental phubbing → Parent–child relationship → PIU	0.05	0.01	0.10
Parental phubbing → BPNS→ PIU	0.05	0.02	0.10
Parental phubbing → Parent–child relationship → BPNS →PIU	0.02	0.01	0.04

*Note N* = 495; ***p <.001; CI: Confidence Interval; PIU: Problematic Internet Use; BPNS: Basic Psychological Needs Satisfaction

Next, multiple mediation analysis was conducted using Model 6 as provided in the PROCESS macro. The results showed that parental phubbing was related to PIU both directly and indirectly (see Table 2; Fig. 1). First, the pathway of ‘parental phubbing → parent–child relationship → PIU’ was significant (indirect effect = 0.05, 95% CI = 0.01 to 0.10). Second, the pathway of ‘parental phubbing → BPNS → PIU’ was significant (indirect effect = 0.05, 95% CI = 0.02 to 0.10). Third, the sequential pathway of ‘parental phubbing → parent–child relationship → BPNS → PIU’ was significant (indirect effect = 0.02, 95% CI = 0.01 to 0.04). Thus, parental phubbing influenced PIU indirectly and was mediated by the parent–child relationship and BPNS, respectively. Moreover, the parent–child relationship and BPNS were mediated sequentially. In other words, parent–child relationship and BPNS play multiple mediating roles in the relationship between parental phubbing and PIU in adolescents.

## Discussion

Given that PIU has become a global social problem among adolescents, it is important to identify the factors associated with it. The rapid development of the Internet not only brings convenience but also causes family-related issues, such as parental phubbing. Whereas prior studies have focused on individual (e.g., social anxiety and self-control) and social factors (e.g., school climate) with parental phubbing and PIU among adolescents [65, 66], the current study stressed family-related problems regarding the association. The findings highlight the importance of the parent–child relationship and BPNS in understanding the relationship between parental phubbing and PIU, which provides valuable insights for prevention and intervention based on family education.

### The multiple mediation model

Consistent with our research hypothesis, parental phubbing was significantly and directly associated with PIU and indirectly associated through the parent–child relationship and BPNS. First, parental phubbing was negatively associated with the parent–child relationship, which influenced PIU. Second, parental phubbing was negatively associated with BPNS, which influenced PIU. Finally, parental phubbing was positively associated with PIU through the sequential mediation of the parental–child relationship and BPNS. These findings indicate that the parent–child relationship and BPNS are important mediating mechanisms.

Our results showed that parental phubbing was positively associated with adolescent PIU. With the diversified development of smartphone functions, an increasing number of people can do work or enjoy entertainment on their phones anytime and anywhere. In China, many adults conduct their work via WeChat or other office software at home after work, and an increasing number of adults play games on their smartphone [67]. Based on this social phenomenon, phubbing behaviours can become considered appropriate and normal by children and adolescents [68]. According to the social learning theory, the behaviour of children and adolescents can be learned and reinforced by their observation of the behaviours of others, especially family members [31, 69]. Therefore, the influence of intergenerational transmission on behaviour is prominent. Consequently, children learn to use smartphones, and this behaviour is reinforced when they experience happiness from smartphone use. Furthermore, the frequent use of mobile phones is generalisable to any medium sharing the same function as mobile phones, such as the Internet. Therefore, parental phubbing is positively related to adolescent PIU.

In line with our assumption, this study showed that the positive association between parental phubbing and adolescents’ PIU was partially mediated by the parent–child relationship. Researchers have proposed that phubbing is a negative phenomenon in social interaction [68], which is connected to a sense of low-warmth and high-rejection in children [70]. Therefore, parental phubbing typically impairs the parent–child relationship in daily family life [41, 71]. Adolescents who experience phubbing receive limited feedback (i.e., responsiveness) from their parents, subsequently leading to insufficient interaction between the parent and children [19, 67]. Adolescents then intentionally spend time on the Internet, such as social media, for entertainment [23, 72, 73]. Thus, parental phubbing can lead to PIU by impairing the parent–child relationship.

In addition, we found that BPNS is a key mechanism through which parental phubbing is connected to PIU. According to SDT, BPNS is an outcome of the influence imposed by a social context, especially the family context [22]. Furthermore, internal motivation encourages individuals to search for a supportive environment to fulfil their unmet needs. Parents frequently being distracted by mobile phones in front of their adolescents can reduce the adolescents’ sense of belonging, which may limit the need for relatedness among adolescents [41]. Additionally, parental phubbing can impede the ability of adolescents to engage in effective face-to-face interactions with their parents, which frustrates autonomy needs [74]. After BPNS is blocked by parental phubbing, adolescents may seek social connections or support from social media to fulfil relatedness needs or use online games to address their needs for autonomy, competence, and relatedness [51, 75]. Individuals are likely to reinforce their Internet use when they feel a sense of satisfaction upon using it. Once adolescents lack sufficient self-control, the likelihood of developing PIU increases [2, 76].

Finally, our research shows for the first time that the parent–child relationship and BPNS work together through multiple mediations. Specifically, the sequential path of ‘parental phubbing → parent–child relationship → BPNS → PIU’ was significant. The result can also be explained by SDT [22] and is consistent with the existing view that meeting basic psychological needs can reduce the likelihood of adolescents developing PIU [23]. Parental phubbing hinders parents in giving timely feedback to their children, which is detrimental to establishing a positive parent–child relationship [20]. Thus, children’s basic psychological needs are not met because the parent–child relationship plays the most important role in their life. To compensate for their basic psychological needs being unmet, children might develop PIU. Specifically, a positive parent–child relationship is important in providing adolescents with a sense of belonging to the family, leading to relatedness satisfaction. In addition, a positive parent–child relationship provides a safe and free environment in which children can make decisions about their own affairs and gain the satisfaction of competence and autonomy. Hence, adolescents with a positive parent–child relationship are unlikely to look for BPNS on the Internet. Therefore, parental phubbing is negatively related to the parent–child relationship and positively related to BPNS, which is negatively related to PIU. Additionally, we did a comparison between parallel model (individually) and multiple model (sequential) in terms of mediating effects (seen supplementary).The results above justify the sequential mediation model. That model, compared with the parallel mediation model, more deeply elucidated how parental phubbing affects adolescents’ PIU and disclosed one more route that parental phubbing affects adolescents’ PIU (through parent–child relationship and BPNS), which can guide us to design more specific interventions to inhibit the PIU of adolescents.

In summary, our study found that parental phubbing worsened the parental–child relationship and hindered adolescent BPNS, making it difficult for adolescents to establish good relations and control their use of the Internet in the real world.

### Limitations and future directions

This study has two limitations. First, a cross-sectional design was used; thus, a causal relationship could not be inferred. As stated by the family system theory, family members mutually influence each other in a bidirectional manner [13]. Therefore, the relationship between parental phubbing and adolescent PIU can also be bidirectional. This phenomenon should be investigated in future studies using a longitudinal design. Second, the data in the present research were collected through self-report measures. According to social cognitive theory, there are differences between people’s perceptions and real objective events [31]. Thus, perceived parental phubbing may be different from parents’ actual behaviour. In future research, the objective measurement method should be adopted, specifically by collecting the frequency with which parents use their mobile phones in front of adolescents and objectively describing or recording the behaviour of parental phubbing. Furthermore, we should examine whether perceived parental phubbing and objective parental phubbing have the same influence on adolescent PIU. Third, this study only included Chinese samples, which limits the generalisation of the findings. Therefore, it is necessary to replicate this study with participants from different cultures.

## Conclusions

In contrast to previous research, the present study tested a multiple mediation model that specified parental phubbing’s association with PIU. From a theoretical point of view, the present research adds to the literature on the relationships among parental phubbing, the parent–child relationship, BPNS, and PIU. Furthermore, the present study extends previous literature by confirming the sequential mediation of the parent–child relationship and BPNS. This is beneficial to understanding how parental phubbing is related to PIU. Moreover, this study offers practical implications. First, the results suggest that parental phubbing may decrease the quality of the relationship between parents and their children and subsequently increase the risk of adolescent PIU. Thus, this finding may be used as a basis to encourage parents to reduce phubbing in front of their children and maintain sufficient face-to-face communication with their children to nurture a strong parent–child relationship. Moreover, the theoretical framework indicated that BPNS serves as an antecedent for PIU, emphasising the need to focus on basic psychological needs for the prevention and intervention of adolescent PIU. Thus, parents and teachers should enhance adolescent awareness of the negative aspects of the Internet from multiple perspectives and create supportive environments to enable adolescents to satisfy their basic psychological needs in real life.

## Electronic supplementary material

Below is the link to the electronic supplementary material.

Supplementary Material 1

## Data Availability

The datasets generated and/or analysed during the current study are available in the Open Science Framework repository, https://osf.io/vtywh/?view_only=ca940e0555c548439ca468ce82de03d0.

## Abbreviations

PIU: Problematic Internet Use
SDT: self-determination theory
BPNS: basic psychological needs satisfaction
CFA: confirmatory factor analysis
CMV: common method bias
