# Correction: Mom, dad, put down your phone and talk to me: how parental phubbing influences problematic internet use among adolescents

**DOI:** 10.1186/s40359-025-02888-6

**Published:** 2025-06-05

**Authors:** Saifang Liu, Peiqian Wu, Xiaoxi Han, Mengyun Wang, Yuecui Kan, Kuiyuan Qin, Jijun Lan

**Affiliations:** 1 School of Psychology, Shaanxi Normal University, 199 South Chang’an Road, Xi’an, 710061 China; 2 Shaanxi Provincial Key Laboratory of Behavior and Cognitive Neuroscience, Xi’an, 710061 China; 3 School of Educational Science, Anhui Normal University, Wuhu, 241000 China; 4 Department of Medical Psychology, Psychological Science and Health Management Center, Harbin Medical University, Harbin, China

**Correction: BMC Psychology (2024) 12:125**

10.1186/s40359-024-01620-0

Following publication of the original article, it was brought to the journal's attention that the article had published without the Methods section. The Methods section has since been added to the published article. The publisher thanks you for reading this erratum and apologizes for any inconvenience caused by this article production error.

