# Supplementary material for: Mom, dad, put down your phone and talk to me: how parental phubbing influences problematic internet use among adolescents
Source: BMC Psychol. 2024 Mar 5;12:125. doi: 10.1186/s40359-024-01620-0 (PMC10916140; doi:10.1186/s40359-024-01620-0)
Supplement: Supplementary file 1 — Supplementary Material 1 [file 40359_2024_1620_MOESM1_ESM.docx]

**SUPPLEMENTARY MATERIAL**

**Testing** **the** **Parallel Mediation Model**

Parallel mediation analysis was conducted using Model 4 as provided in the PROCESS macro. The results showed that parental phubbing was related to PIU both directly and indirectly (see S Table 1 & S Figure 1). First, the pathway of “parental phubbing → parent–child relationship → PIU” was significant (indirect effect = .05, 95% CI = .01 to .10). Second, the pathway of “parental phubbing → BPNS → PIU” was significant (indirect effect = .07, 95% CI = .03 to .11).


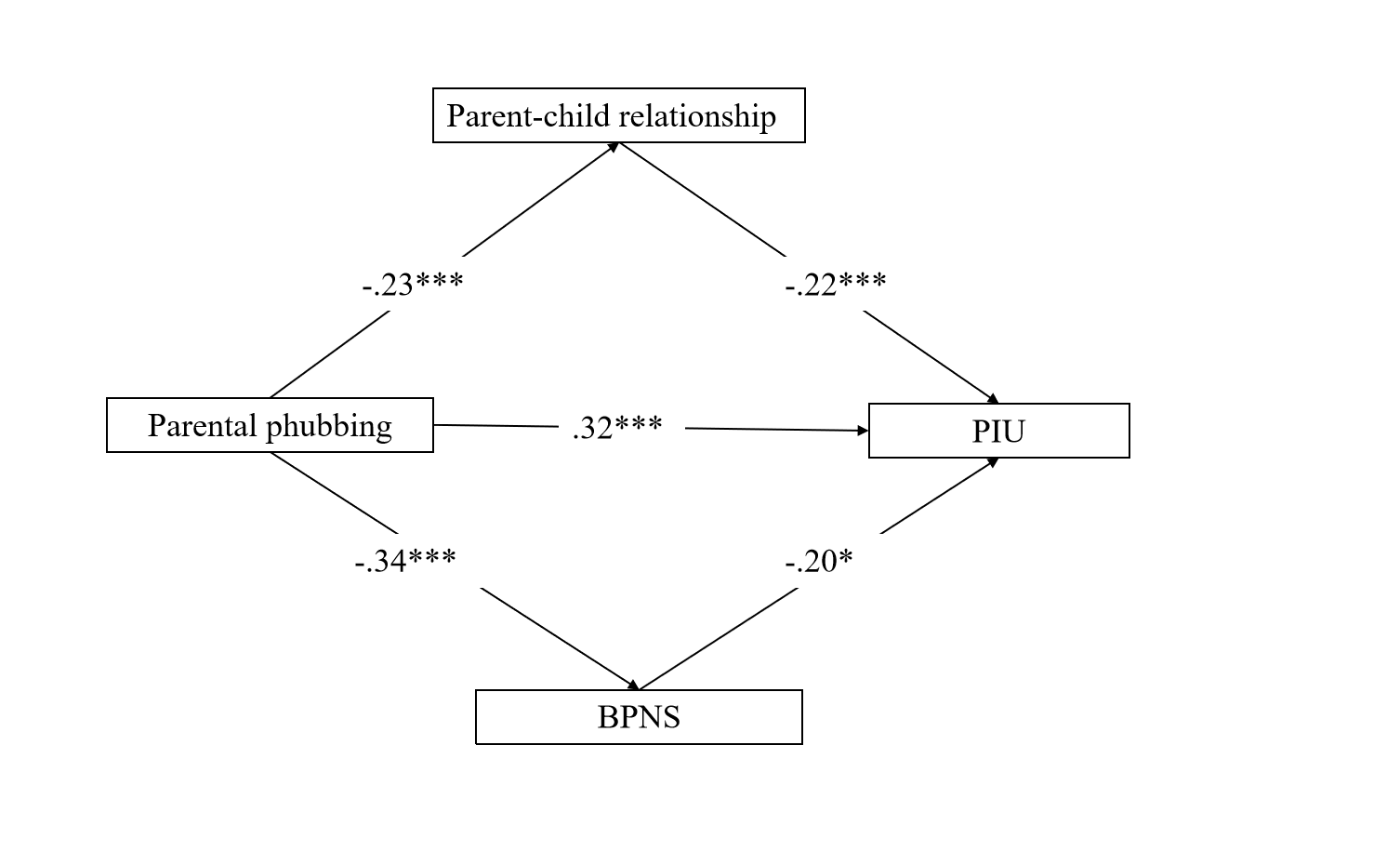


S Figure 1. The parallel mediation model. Path values show path coefficients. ****p* < .001, ***p* < .01.

S Table 2. Testing the Pathways of the Parallel Mediation Model

| Path | Point  estimate | 95% CI | |
| --- | --- | --- | --- |
|  |  | Lower | Upper |
| Direct effects |  |  |  |
| Parental phubbing 🡪 PIU | .32*** | .18 | .46 |
| Parental phubbing 🡪 Parent–child relationship | -.23*** | -.30 | -.15 |
| Parent–child relationship 🡪 PIU | -.22*** | -.38 | -.06 |
| Parental phubbing 🡪 BPNS | -.34*** | -.46 | -.21 |
| BPNS 🡪 PIU | -.20*** | -.30 | -.11 |
| Indirect effects |  |  |  |
| Parental phubbing 🡪 Parent–child relationship 🡪 PIU | .05 | .01 | .10 |
| Parental phubbing 🡪 BPNS🡪 PIU | .07 | .03 | .11 |

*Note*. *N* = 495. ****p* <.001.
Abbreviations. CI: Confidence Interval; PIU: Problematic Internet Use; BPNS: Basic Psychological Needs Satisfaction
